# Cardiovascular magnetic resonance follow-up of the Marfan's thoracic aorta after personalized external aortic root support surgery

**DOI:** 10.1186/1532-429X-16-S1-P116

**Published:** 2014-01-16

**Authors:** Cemil Izgi, Evangelia Nyktari, Raad Mohiaddin

**Affiliations:** 1 Royal Brompton Hospital, London, UK; 2 National Heart and Lung Institute, Imperial College, London, UK

## Background

Recently, personalized external aortic root support (PEARS) has been proposed as an alternative prophylactic surgery to prevent dilatation of the aortic root and dissection in Marfan syndrome patients. Briefly, a model of each patient's aortic root and ascending aorta is created based on cardiac magnetic resonance (CMR) images. The model is then used to produce a bespoke external aortic root support made up of polymer mesh for each patient ready for surgical implantation[1]. The surgery is quicker than conventional aortic root replacement without the need for cardiopulmonary bypass or circulatory arrest. In addition, the blood endothelium interface is not interrupted and the native aortic valve is preserved obviating the need for life-long anticoagulation. Early positive result have been acknowledged in a recent NICE guidance document[2,3]. The aim of this study is to assess long term changes in aortic dimensions following PEARS surgery.

## Methods

27 patients with Marfan syndrome had PEARS surgery between 2004 and 2012 at the Royal Brompton Hospital. 24 of these patients had regular follow-up CMR examinations before and after surgery and formed the study group. Aortic size measurements were as follows: 1- aortic annulus diameter, 2- three commissure to cusp diameters of sinus Valsalva measured from a transverse section of the aorta at the level of aortic valve leaflet closure, 3- area of sinus Valsalva, 4- ascending and 5- descending aorta diameters at the level of right pulmonary artery and 6-aortic arch diameter. Aortic size measurements immediately before surgery and at the latest follow-up were compared. All measurements were performed randomly and blinded

## Results

**Figure 1:**
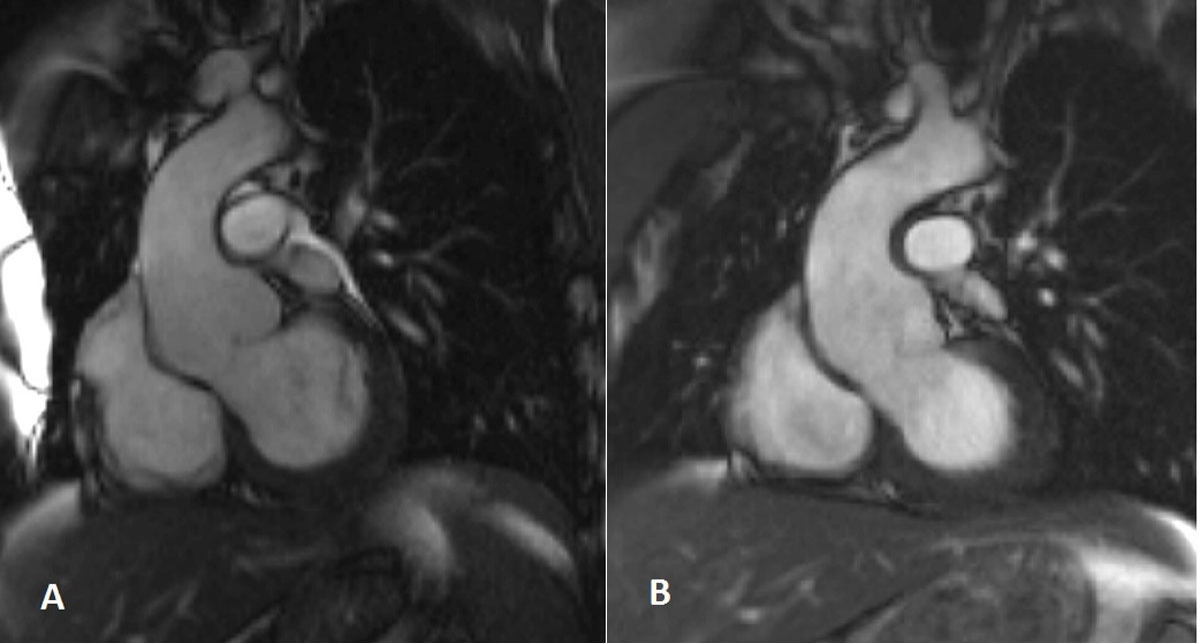
**Corresponding diastolic frames selected from complete cine acquisition of the aortic root and ascending aorta acquired in the same patient immediately before (A) and 4 years after surgery (B)**. Aortic wall is thickened at the site of surgery but no increase in the size of aortic root and ascending aorta.

The follow-up studies were performed 51.6 ± 26.4 months after surgery (median 50.5 months, range 8-101 and interquartile range 25.5-72 months). The results are tabulated in Table 1. There was a slight but significant decrease in commissure-cusp diameters in follow-up (mean -0.8 ± 2.5 mm, range -7 - +3 and IQ range -3 - +1 mm), though the other index of aortic root size, sinus Valsalva area was unchanged. Hence the aortic root size was held stable without any significant increase in follow-up after PEARS surgery (Figure 1). There was no significant change in aortic annulus, ascending aorta and aortic arch diameters. However there was slight but significant increase in descending aorta diameter.

**Table 1:** Aorta measurements before and after PEARS surgery

	Pre-op	Last follow-up	P (Paired t test)
Sinus Valsalva area (cm2)	16.3 ± 1.9	15.7 ± 2.7	0.1

Sinus Valsalva, mean diameter (mm)	43.5 ± 2.3	42.7 ± 3.7	0.1

All sinus Valsalva diameters†	43.5 ± 2.65	42.7 ± 3.95	0.01*

Sinus Valsalva, largest diameter (mm)	44.9 ± 2.8	44.0 ± 3.9	0.12

Ascending Aorta diameter (mm)	32.3 ± 3.7	32.6 ± 3.7	0.5

Annulus diameter (mm)	28.9 ± 2.2	28.7 ± 2.4	0.6

Aortic Arch (mm)	24.1 ± 2.1	23.8 ± 3.1	0.6

Descending Aorta diameter (mm)	22.6 ± 2.5	23.5 ± 2.9	0.01*

† comparison of mean of all individual commissure-cusp diameters (3 × 24 = 72 diameters)

## Conclusions

This study demonstrates the long term effectiveness of PEARS surgery in preventing progressive aortic root dilatation in Marfan syndrome patients.

## Funding

None.
